# Photoacoustic characteristics of carbon-based infrared absorbers

**DOI:** 10.1016/j.pacs.2021.100265

**Published:** 2021-05-19

**Authors:** Jussi Rossi, Juho Uotila, Sucheta Sharma, Toni Laurila, Roland Teissier, Alexei Baranov, Erkki Ikonen, Markku Vainio

**Affiliations:** a Photonics Laboratory, Physics Unit, Tampere University, Tampere, Finland; b Patria Aviation Oy, Tampere, Finland; c Metrology Research Institute, Aalto University, Espoo, Finland; d IES, University of Montpellier, CNRS, 34095, Montpellier, France; e VTT MIKES, Espoo, Finland; f Department of Chemistry, University of Helsinki, Helsinki, Finland

**Keywords:** Candle soot, Carbon nanotubes, Photoacoustic response

## Abstract

We present an experimental comparison of photoacoustic responsivities of common highly absorbing carbon-based materials. The comparison was carried out with parameters relevant for photoacoustic power detectors and Fourier-transform infrared (FTIR) spectroscopy: we covered a broad wavelength range from the visible red to far infrared (633 nm to 25 μm) and the regime of low acoustic frequencies (< 1 kHz). The investigated materials include a candle soot-based coating, a black paint coating and two different carbon nanotube coatings. Of these, the low-cost soot absorber produced clearly the highest photoacoustic response over the entire measurement range.

## 1 Introduction

In addition to its many applications in spectroscopy [[1], [2], [3], [4]] and imaging [5,6], the photoacoustic (PA) effect is useful for electromagnetic power detection due to its wavelength independency and high detection sensitivity. In a typical photoacoustic optical power detector, the incident radiation is first modulated by a chopper and then directed through a window to a PA cell. The cell contains an optical absorber to generate an acoustic wave at the chopping frequency. It is filled with gas that carries the acoustic signal to a sensitive microphone, whose output is proportional to the optical power incident on the detector. An example of a commonly used PA detector is Golay cell, in which the signal is recorded by optical readout of a thin reflective membrane that stretches due to the acoustic wave [7]. Although the photoacoustic detection principle works at practically any wavelength, it is mainly used in the infrared and terahertz (THz) regions, where it is one of the most sensitive power detection methods available [[8], [9], [10], [11]].

An essential component of the PA power detector is the absorber. An ideal (hyperblack) absorber would have a flat and perfect (100 %) absorbance at all wavelengths [12]. The broad and uniform spectral responsivity is important not only for general-purpose power detectors but also from the metrological point of view: Traceable power measurements in the infrared and THz regions benefit from the possibility of transferring the calibration to the visible wavelength region, where a more accurate responsivity scale is available [13]. Another example of an application that requires an optically broadband absorber is Fourier Transform Infrared (FTIR) spectroscopy, where photoacoustic detection is often used due to its background free nature, broad wavelength coverage and large dynamic range. Highly absorptive carbon reference materials are used to normalize FTIR spectra of unknown samples [14]. In other words, the spectrum of an unknown sample is measured and divided by the FTIR spectrum of the reference absorber. This procedure removes the spectral dependence of the FTIR instrument if the reference absorber has a uniform and/or well-characterized spectral responsivity throughout the whole measurement range [15].

While the emissivities of different black materials have been extensively investigated [[16], [17], [18]], information about their photoacoustic properties is difficult to find in the literature. Detailed studies of PA efficiencies of different carbon-based absorbers in the visible wavelengths have been published in view of ultrasound generation for medical applications [19,20]. However, as far as we know, comparisons of photoacoustic properties of absorber materials for conditions relevant in optical power detection and FTIR measurements are yet to be reported. These applications require information about photoacoustic performance of different materials at low acoustic frequencies (from approximately 10 Hz to 1 kHz) and over a wide range of wavelengths, particularly in the infrared part of the spectrum.

In this paper, we report an experimental comparison of the photoacoustic responsivities of different carbon-based absorbers over a wide wavelength range, from the visible red to far infrared (25 μm). The results of this research benefit the development of next-generation photoacoustic power detectors, traceable long-wavelength power measurements, as well as photoacoustic FTIR spectroscopy. We have also studied the dependence of signal strength on modulation (chopping) frequency and acoustic carrier gas for our experimental setup, which is based on a silicon cantilever microphone. The cantilever-enhanced photoacoustic method has already led to some of the best detection sensitivities in photoacoustic trace-gas spectroscopy [3,4,21], and it has the potential to significantly advance optical power detector development as well.

## 2 Photoacoustic instrument and absorber materials

The experiments described in this paper were carried out using a commercially available photoacoustic detector (PA301, Gasera Ltd). The detector is originally designed as an accessory for FTIR analysis of solid and liquid samples. The incoming optical power is first modulated with a chopper and then collected with a gold-coated ellipsoid mirror. The mirror guides the light beam through the KBr window of the photoacoustic cell and to the center of the sample, which in our case is the absorber under study.

The instrument works over a wide wavelength range that is fundamentally limited by the transmittance of the KBr window (0.3–25 μm). In practice, the short wavelength side is cut to 1.5 μm due to the FTIR beam splitter, which contains germanium.

The acoustic signal generated at the absorber is recorded with a cantilever microphone fabricated from silicon [22]. The cantilever is designed for the detection of low acoustic frequencies (<1 kHz), where it shows a large linear dynamic range and high detection sensitivity [23]. The photoacoustic cell is filled with an acoustic carrier gas, which in our measurements is typically air, N_2_ or He. The gas pressure is the same as the ambient pressure. The absorber under study is placed in a 10-mm diameter sample cup made of aluminum. The sample cup is located behind the window of the photoacoustic cell.

### 2.1 Absorbers

**Fig. 1:**
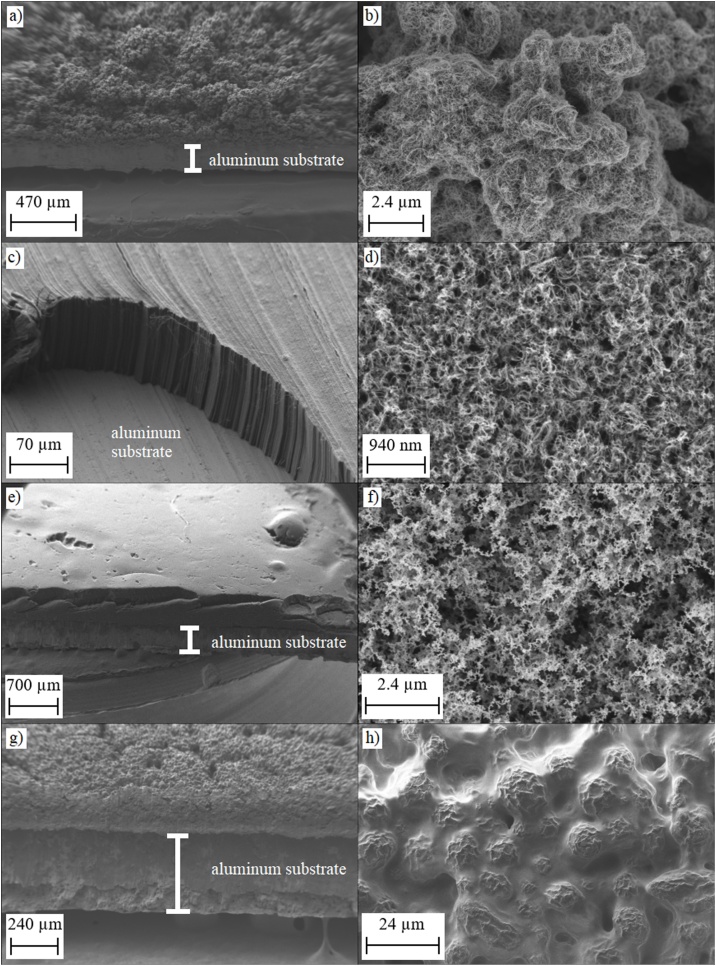
Side view (left row) and top view (right row) SEM pictures of the cut samples. a) – b) S-VIS, c) – d) Vantablack, e) – f) candle soot and g) – h) Nextel-coating.

The photoacoustic comparison was carried out with a set of black absorbers that potentially work both in the visible and infrared parts of the electromagnetic spectrum. As relevant prior information of the photoacoustic properties of different absorbers is scarce, we selected the samples mainly based on the previous studies concerning the emissivity, reflectivity and ultrasonic photoacoustic conversion efficiency of various black materials [[16], [17], [18], [19], [20],[24], [25], [26]]. The selected absorbers include two commercially available carbon nanotube (CNT) surfaces, candle soot and a commercially available ultrablack paint (Nextel), all of which are briefly described below. Each absorber was fabricated on an aluminum substrate. The absorber thicknesses were determined by scanning electron microscope (SEM) measurements, see Fig. 1. The absorber parameters are summarized in Table 1.

**Table 1:** Absorber properties, including emissivity (with wavelength ranges), fabrication method and the measured thickness of the film.

Absorber	Soot	S-VIS	Vantablack	Nextel
Emissivity	0.68−0.99 [33]	0.994−0.999 [34]	0.98−0.99 [18]	0.971 [26]
	(0.38−16 μm)	(3−14 μm)	(5−12 μm)	(5−20 μm)
Fabrication method	Candle flame	Spray coating	CVD	Spray coating
Thickness	360−510 μm	200–480 μm	80−90 μm	180−220 μm

Carbon nanotube coatings were chosen for the comparison because they are known to have low reflectances from the visible to the infrared region. In addition, vertically aligned CNT arrays have provided excellent performance in thermopile and pyroelectric thermal detectors [12,24,27,28]. Our CNT absorbers were ordered from Surrey Nanosystems Ltd. Two different samples were selected: Spray-applied coating of randomly oriented CNTs (S-VIS, Fig. 1a and b) and a surface that consists of vertically aligned CNT arrays (Vantablack, Fig. 1c and d). The average thicknesses of the absorbing CNT layers are approximately 85 μm and 340 μm for the Vantablack and S-VIS samples, respectively. The manufacturer has fabricated the absorbing layers on aluminum substrate, which were cut to fit in the 10 mm sample cup of our PA detector. Extreme care was taken to not to touch the fragile sample surfaces.

**Fig. 2:**
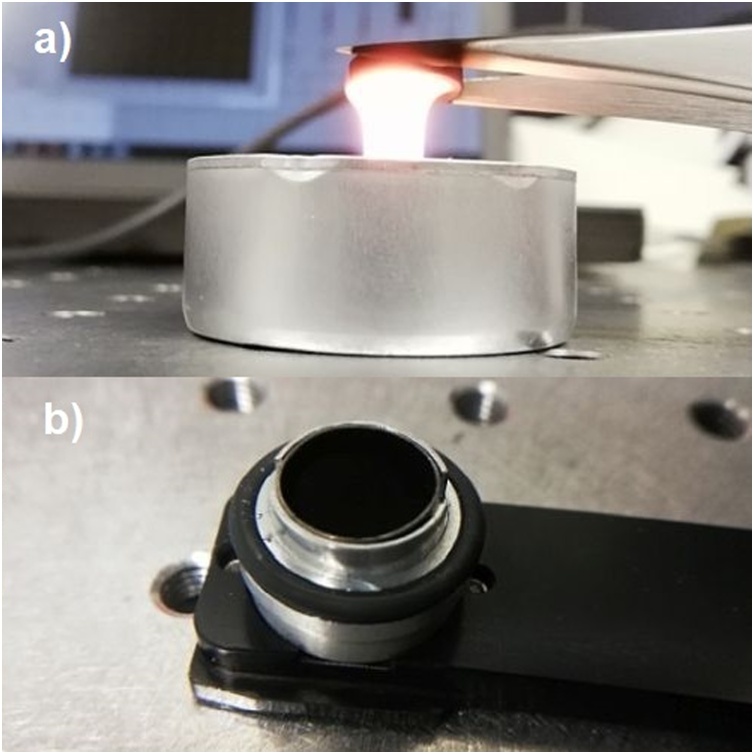
a) Sooting process, where a sample cup is held in the tip of a candle flame. b) A sample cup and holder of the PA301 photoacoustic detector.

Among other applications, candle soot coatings have been used in efficient pyroelectric energy conversion [16,17] and ultrasound generation [19]. Although the high specific heat capacity of carbon-based soot and paint coatings is not necessarily ideal for other thermal detectors [25], they are potentially useful materials in photoacoustic detection. Our soot absorbers (Fig. 1e and f) were fabricated directly on the bottom of the aluminum sample cup using a paraffin wax candle flame (Fig. 2a). The average thickness of the soot surface is 435 μm, as verified by the SEM measurements. This simple method is suitable for reproducible synthesis of uniform layers of carbon nanoparticles, the layer thickness depending on the deposition time [19]. The combustible material of candle wax is for the most part paraffin (C_n_H_2n+2_) and the burning process proceeds upwards in gravitational environment if adequate amount of oxygen is available. If the process is interfered by cooling the tip of the flame, the candle starts smoking and due to the incomplete burning process soot particles consisting of cyclic, highly unsaturated, polycyclic aromatic structural elements ((C_3_H)_n_) are formed [29]. The primary soot particles grow through agglomeration, dehydration, and coagulation to as much as a few million carbon atoms and are deposited to the surface of a sample cup positioned at the tip of the flame (Fig. 2a).

The Nextel paint (Velvet-Coating 811-21, Mankiewicz Gebr. & Co) was included in the comparison because of its high emissivity over the wavelength range investigated in this work [26]. A painted surface is also more robust compared to the soot and nanotube samples which get easily damaged in contact with fingers or tools. The paint was applied directly on the surface of a sample cup by a professional painter by spraying with the required instruments and technique instructed in the datasheet of the manufacturer (Fig. 1g and h). The average thickness of the Nextel coating is 200 μm.

In summary, the SEM measurements confirm that the Vantablack surface is highly uniform (Fig. 1c) as expected [[30], [31], [32]]. Its nanotube structure can be clearly distinguished in Fig. 1d. The S-VIS surface (Fig. 1a) is much rougher, but the magnification (Fig. 1b) reveals its nanotube nature. Soot surface is very smooth (Fig. 1f), with a surface roughness similar to that of Vantablack. The painted Nextel surface (Fig. 1h) is the most sealed one of the samples investigated here, leading to a reduced effective surface area. This is a likely explanation of Nextel surface’s modest photoacoustic conversion efficiency, as discussed in the following section.

## 3 Measurements and results

The photoacoustic responsivities of the selected absorbers were compared over a broad spectral range, from the visible red (633 nm) to far infrared (25 μm). These comparisons were done using two complementary approaches. First, we measured the relative photoacoustic responsivities at several discrete wavelengths using monochromatic continuous-wave lasers. Second, similar measurements were carried out with an FTIR spectrometer that is equipped with a broadband incandescent light source. The FTIR measurements allowed us to extend the comparison to wavelengths inaccessible with lasers. In order to cancel out instrumental effects, all measurements were compared against the best-performing absorber, which in our case turned out to be the one based on candle soot.

### 3.1 Laser measurements

**Fig. 3:**
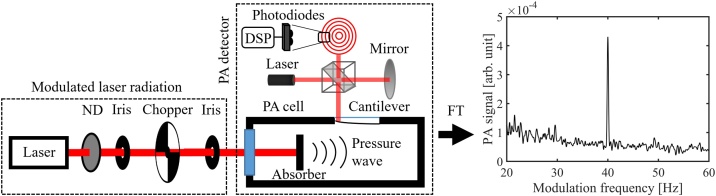
Simplified schematic of the photoacoustic laser power measurement setup (not all mirrors are shown). The chopped laser beam is aligned to the photoacoustic cell and the acoustic signal is recorded with the cantilever microphone by highly sensitive interferometric readout and processed with a Digital signal processor (DSP). The laser power level is adjusted with a neutral density filter (ND). The right-hand side of the figure shows an example of a PA spectrum, measured with 40 Hz chopping frequency, 6.26 s Fourier time constant and with an optical power of 50 nW (at 633 nm wavelength).

The setup used for laser measurements is shown schematically in Fig. 3. The laser power was modulated with a rotating-disk chopper before directing the laser beam to the absorber under study in normal-incidence configuration. The field of view seen by the detector was limited by irises to avoid any background radiation to be summed in the modulated laser radiation. In all measurements, the laser beam was collimated and directed to the center of the absorber under study with a beam diameter smaller than 3 mm. Unless otherwise mentioned, the laser power levels were set to the same value (1.82 mW) by adjusting the laser drive current and/or neutral density filters. A very high signal-to-noise ratio (SNR) of over 10^4^ was obtained with this power level in the measurements reported here.

**Fig. 4:**
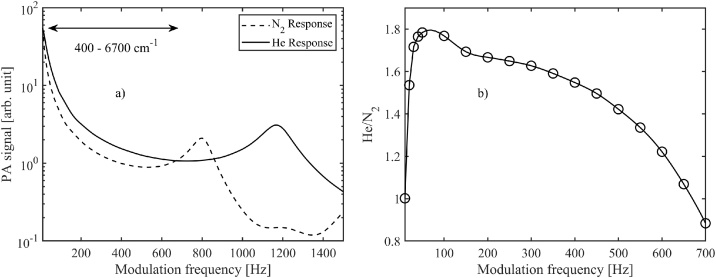
a) Photoacoustic response curves recorded with a 9.2 μm laser, candle-soot absorber and with two different carrier gases, He and N_2_. The arrow indicates the wavenumber range covered in the complementary FTIR measurements (see next section and the Appendix). b) The ratio of these two curves in the frequency range of 10 to 700 Hz.

**Fig. A1:**
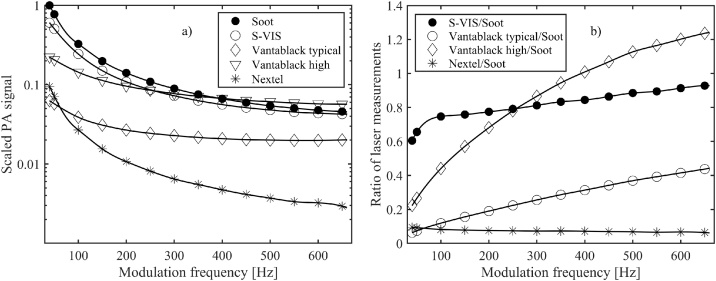
The photoacoustic response curves recorded with a 14.85 μm laser for all the absorbers in a). In b) ratios with respect to soot are presented. Carrier gas helium and modulation frequency range of 40 Hz to 650 Hz.

The photoacoustic signal recorded by the cantilever microphone was digitized, and the time-domain signal was subsequently Fourier transformed (FFT, Fast Fourier Transform) in real time to get the PA signal spectrum [15,35]. The actual signal proportional to the incident optical power was then obtained from the spectrum as the maximum value of the peak at the chopping frequency, using a recording time (Fourier time constant) of 1.57 s, unless otherwise mentioned. An example of a Fourier-transformed PA signal is shown in the inset of Fig. 3. The FFT method was used instead of lock-in detection in order to obtain the full information of the photoacoustic spectrum. The photoacoustic response depends on the chopping frequency, as illustrated in Fig. 4a for the candle soot-based absorber. (Similar plots for the other absorbers are presented in Fig. A1a of the Appendix). Note that this modulation-frequency dependence includes contributions from different parts of our PA301 photoacoustic detector, not just the absorber [15]. As an example, the figure clearly shows a mechanical resonance peak of the silicon cantilever. Although the cantilever microphone can be used with any modulation frequency within the range presented in Fig. 4, the best measurement SNR is typically obtained with frequencies below 100 Hz. At low frequencies below 20 Hz, the noise due to the external vibrations dominates. At higher frequencies the fundamental limit is set by the Brownian motion of the gas molecules when all external and electrical noise sources are eliminated [15]. For example, with soot at 40 Hz, the noise-equivalent power was found to be in the range of 10 nW/√Hz depending on the laser wavelength

Both the cantilever resonance frequency and the strength of the photoacoustic signal depend on the acoustic carrier gas. Due to its high thermal conductivity, helium is known to be one of the best choices in terms of signal maximization [36,37] and this was confirmed in our measurements. As an example, with the candle-soot absorber, helium gives up to 80 % larger signal than nitrogen, depending on the modulation frequency (Fig. 4b). The same tendency is observable also with the other absorbers investigated here – see Fig. 10. The largest He/N_2_ enhancement factor (of about 2.5) was obtained with the Nextel-painted surface.

**Fig. 5:**
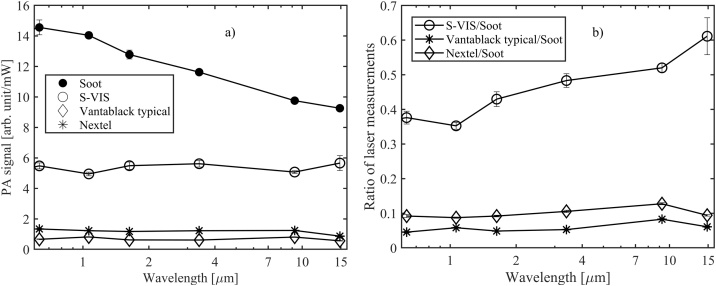
a) The photoacoustic signals of different absorbers normalized to 1 mW of optical power. b) The same spectral responsivities divided by that of the candle soot absorber, as measured with monochromatic lasers at six different wavelengths. The lines between the measured points are guides to the eye and do not present any physically meaningful fitting function. The chopping frequency was 40 Hz, and the acoustic carrier gas was helium.

**Fig. 6:**
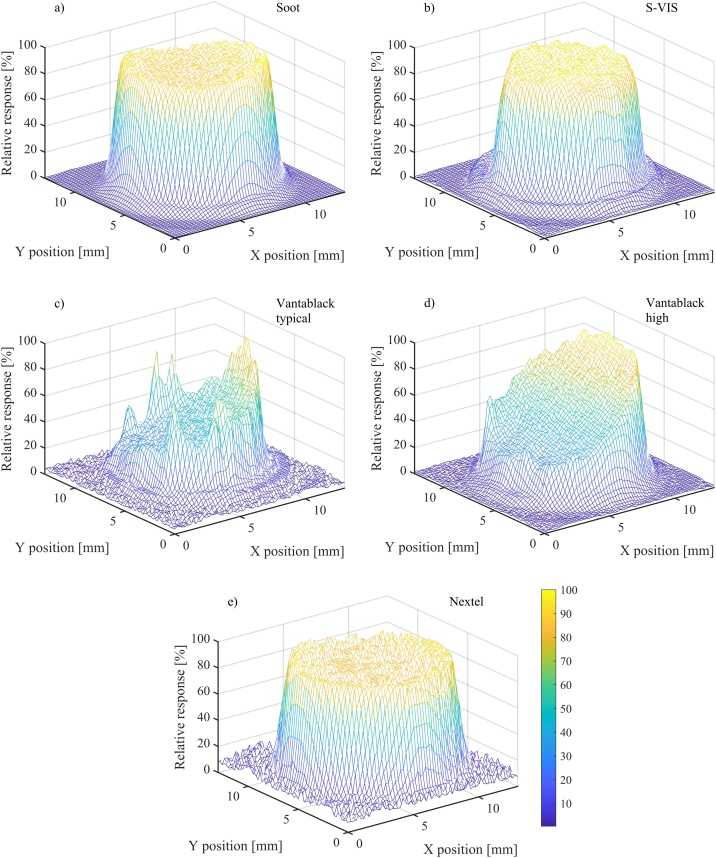
Spatial uniformity of PA response for different absorbers. The laser wavelength and the probe laser spot size were 633 nm and 1.2 mm, respectively.

After characterizing the modulation-frequency dependence of the photoacoustic response, we compared the PA signals of the different absorber materials at six different wavelengths: 633 nm (He-Ne laser), 1064 nm (Yb-fiber laser), 1.63 μm (diode laser), 3.39 μm (He-Ne laser), 9.24 μm (Quantum Cascade Laser, QCL) and 14.85 μm (QCL). All lasers are continuous-wave lasers that produce highly monochromatic light. Other lasers are commercially available, but the 14.85 μm quantum cascade laser was custom-made for this work [38,39]. The result of this comparison is presented in Fig. 5a, which shows the absolute spectral responses of different absorbers with a chopping frequency of 40 Hz. Fig. 5b shows the spectral responsivities divided by that of the candle-soot absorber, which gives clearly the strongest PA signal at all wavelengths. The error bars represent the estimated combined standard uncertainties, the dominant uncertainty sources being the reference power meter calibration and measurement repeatability (statistical measurement uncertainty). The uncertainty stemming from repeatability dominates at longer wavelengths and power calibration uncertainty dominates at short wavelengths. The power-meter dependent total uncertainty of calibration is 1–5 %, including reference meter calibration uncertainty and spatial nonuniformity uncertainty if provided by the manufacturer. The uncertainty due to measurement repeatability is 1–8 % depending on the absorber and measurement wavelength. This includes the effect of spatial nonuniformity of PA response (see Fig. 6) which was minimized by guiding the laser beam to the center of the absorber.

### 3.2 Spatial uniformity of PA response

The spatial uniformity of photoacoustic response for each absorber was measured by scanning the absorbing area with a 633 nm He-Ne laser. The laser was attached to a motorized xy-translation stage and the laser spot size was 1.2 mm (1/e^2^ diameter). The xy-scanner step size and the laser beam chopping frequency were 0.2 mm and 200 Hz, respectively.

**Fig. A2:**
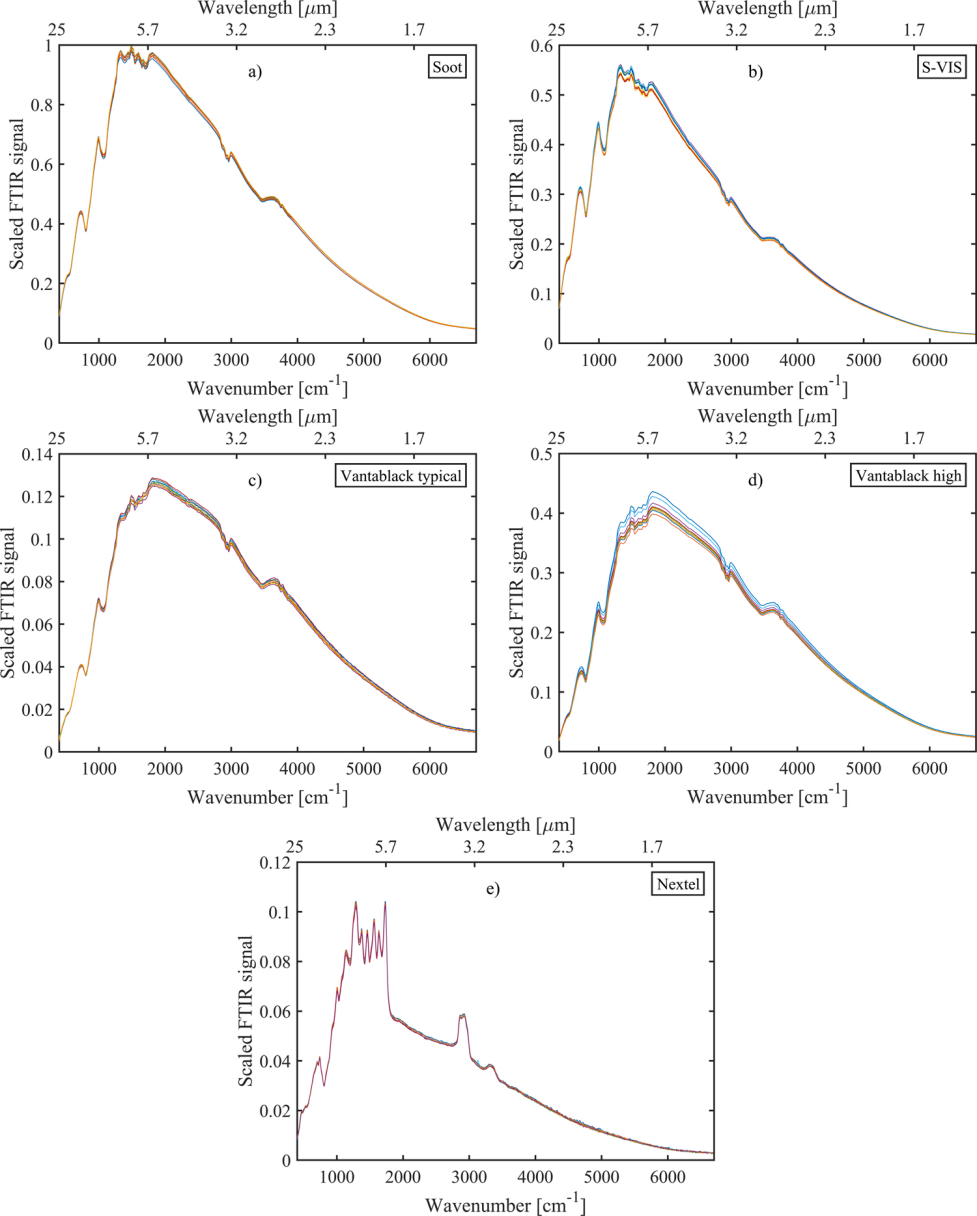
Photoacoustic FTIR spectra recorded with different absorbers. These data were used to calculate the average FTIR spectra and statistical uncertainties in Fig. 8, Fig. 9 of the main text. Carrier gas was helium.

The results in Fig. 6 show large spatial variations with Vantablack samples (Fig. 6c and d), partly explaining the higher uncertainty in measurement repeatability, see for example Fig. A2c and d of the Appendix. Soot, S-VIS and the Nextel surfaces are more uniform, and the relative PA responses are between 85 and 100 %. Both Nextel and soot have higher PA responsivities at the edges. Both the edge effect and high nonuniformity of Vantablack absorbers have also been reported in pyroelectric spatial uniformity measurements [[40], [41], [42]].

### 3.3 FTIR measurements

**Fig. 7:**
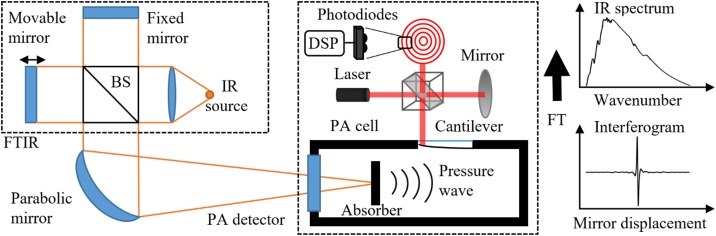
The principle of photoacoustic characterization of different absorbers using an FTIR spectrometer. Inside the FTIR, a broadband light emitted by the IR source (SiC) is modulated by the movable mirror of the interferometer. The collimated output is focused into the photoacoustic cell and the acoustic signal is recorded with the cantilever microphone by highly sensitive interferometric readout. Fourier transform (FT) of the interferogram gives the photoacoustic spectrum.

The longest wavelength accessible in the laser measurements was 14.85 μm, which was achieved with our state-of-the-art quantum cascade laser technology. In order to extend the PA comparison to even longer infrared, we repeated the measurements with another setup (Fig. 7). The spectral range from 1.5–25 μm (6700–400 cm^−1^) was continuously covered using a SiC thermal light source combined with an FTIR spectrometer (Bruker IRCube Matrix M series). The light emitted by the SiC was passed through the scanning Michelson interferometer of the FTIR instrument, thus producing a modulated output that was analyzed with the PA301 photoacoustic detector. As one of the mirrors of the Michelson interferometer is scanned at a constant speed *u* the optical power component at wavelength $\lambda_{o}$ at the interferometer output is sinusoidally modulated at frequency$\;f=2u/\lambda_{o}=\left(2u/c\right)\nu_{o},$ where $\nu_{o}$ is the optical frequency and *c* is the speed of light. In our case of broadband light source, the interferogram (see the inset of Fig. 7) is the sum of the modulated signals of all wavelengths [43]. In other words, each optical frequency of the broadband light source is unambiguously mapped to a different acoustic frequency, and the Fourier transformed output of the PA301 detector (PA spectrum) is a down-converted replica of the original optical spectrum weighted by the spectral dependency of the PA detector, including the absorber. (The respective PA spectrum with optical wavelength on the horizontal axis can be recovered from the down-converted spectrum by multiplying the inverse of the acoustic-frequency axis by 2u). We have chosen the FTIR mirror scanner speed ($u=5.064\times 10^{-4}\;\text{m}/\text{s}$) such that the optical spectrum of the SiC light source is mapped to acoustic frequencies between 40.5 and 679 Hz, see the Appendix for details. This acoustic frequency range is well below the resonance frequency of the PA detector as indicated in Fig. 4a.

**Fig. 8:**
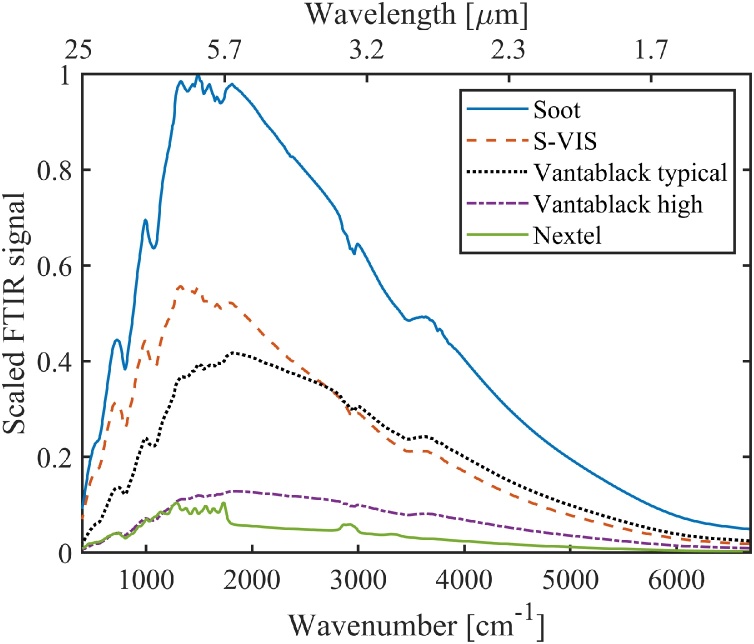
Photoacoustic FTIR spectra of different absorbers. All spectra are scaled by dividing them with the maximum signal of the soot sample. The spectral shape is mostly due to the SiC light source, whose emission spectrum closely follows Planck’s law. The long-wavelength side of the spectrum is attenuated due to the increased losses of the FTIR’s KBr beamsplitter at > 20 μm. The dips in the spectra are caused by absorbing molecules in the light path (mostly water vapor in the laboratory air). The spectral resolution of the FTIR instrument was set to 15 cm^−1^, and the acoustic carrier gas used in the measurements was helium. For Vantablack, two curves are shown to exemplify the significant sample-to-sample variation, see text for details.

**Fig. 9:**
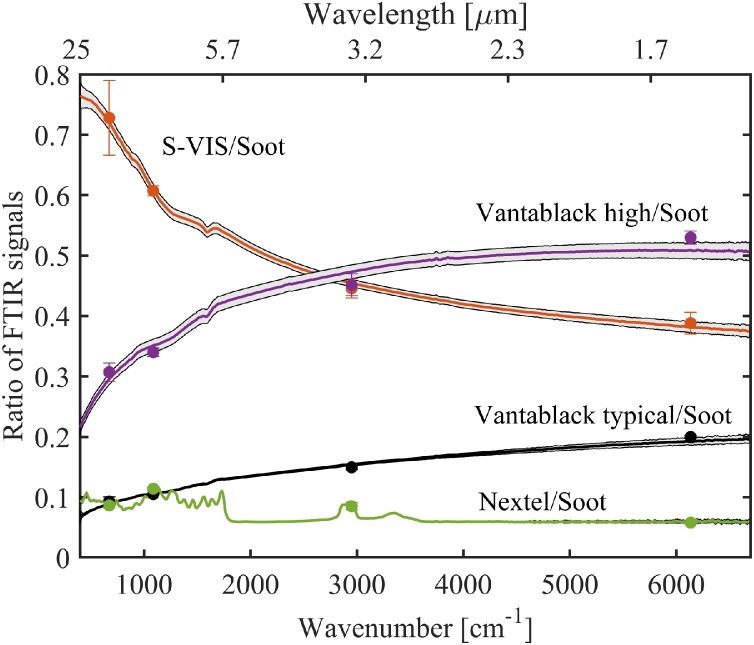
Photoacoustic signals of different absorbers divided by that of the candle soot absorber, as calculated from the PA FTIR spectra of Fig. 8. The shaded areas around the curves describe the statistical uncertainties of the FTIR measurements. Reference measurements done with lasers are indicated by dots and their statistical uncertainties by error bars.

The photoacoustic FTIR spectra of different absorbers are presented in Fig. 8. Again, to cancel out the instrument function of the measurement setup (spectral variations of the light source, beam splitters, mirrors, etc.), we divided these spectra with that of the best absorber (candle soot) to get the relative PA responsivities as a function of optical wavelength. These ratios are shown in Fig. 9. Note that the individual photoacoustic FTIR spectra include contributions of the acoustic-frequency dependencies of the detector and the absorbers, because each optical frequency corresponds to a different acoustic frequency in the FTIR spectrum. In order to assist the interpretation of the FTIR measurements, we have plotted the modulation-frequency dependencies of different absorbers in the Appendix (Figs. A1a-b). Figure A1a indicates that the influence of modulation frequency on the S-VIS/Soot and Nextel/Soot ratios of Fig. 9 is small (except for the very long wavelengths), but the Vantablack/Soot ratios are strongly affected by this effect. The FTIR results were further validated with laser measurements, which are indicated by dots in Fig. 9. The laser measurements were carried out at modulation frequencies that correspond to the FTIR modulation frequencies of the respective optical wavelengths (see the Appendix Table A1).

**Fig. 10:**
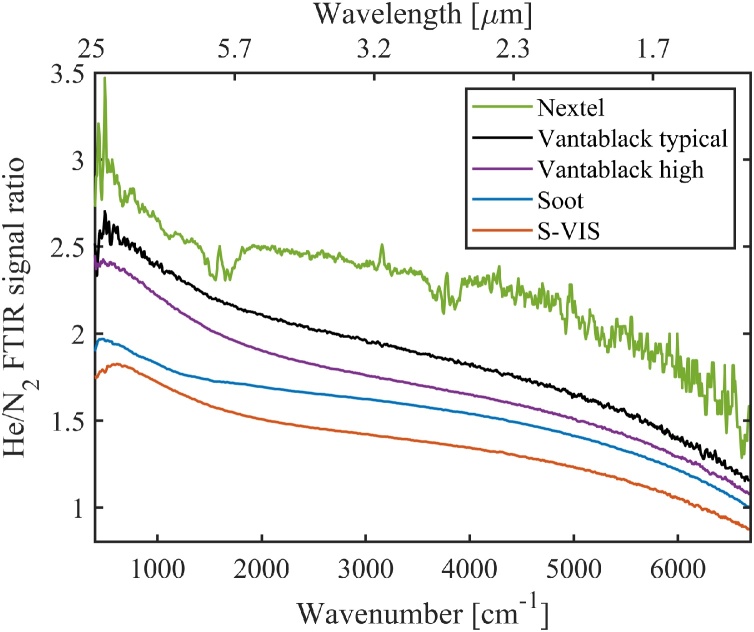
The ratios of photoacoustic FTIR signals measured with two different acoustic carrier gases, He and N_2_.

Despite the added complexity due to varying modulation frequency, the photoacoustic FTIR spectra give valuable complementary information to the laser measurements. As an example, Fig. 8 reveals that the spectral responsivities of other absorbers are smooth, but the Nextel coating loses its flat absorptivity above ∼2.8 μm and starts to act like a molecular absorber. The peak around 3 μm is a signature of the OH-group and the peaks between 3.3 and 3.6 μm and above 5 μm are mostly due to hydrocarbon molecular vibrations of the paint substances [16] (see Fig. A2e of the Appendix for a more detailed plot). It is also worth noting that the gradual improvement of the photoacoustic FTIR response of the vertically aligned CNT absorber (Vantablack) towards shorter wavelengths does not imply a real wavelength dependency, but rather reflects the improvement of Vantablack’s responsivity with increasing modulation frequency (Fig. A1 b).

The shaded areas around the curves in Fig. 9 describe the statistical uncertainties of the ratio measurements, as estimated from the standard deviations (1σ) of the FTIR measurements of each absorber. In order to calculate the standard deviations, the spectra were recorded 10 times for each sample. The raw data are presented in the Appendix, Fig. A2. The relative standard uncertainties are below 5 % for all the ratios over the entire measurement range – the increase of the uncertainty towards the edges of the spectral range is mostly due to the decreased spectral intensity of the light source at the detector (Fig. 8).

We also tested the reproducibility of sample preparation by producing and measuring multiple samples. With the candle-soot and S-VIS absorbers (5 of each), the maximum differences between the lowest and highest responsivities were 10 % and 15 % compared to the highest PA responsivity, respectively. Nextel surfaces were painted the same way and at the same time, and the differences between three different samples was less than 2 %. The highest variation was observed between different Vantablack samples, in which case “the best cut” gave 2.7 times higher PA signal than the worst one. This is illustrated in Fig. 8, which shows the FTIR curve of a typical Vantablack sample along with the best one. Large variations in PA responsivities are also observed in spatial uniformity measurements (Fig. 6). The reasons for such large variations between different Vantablack samples are currently unknown. However, a possible explanation seems to be that it is practically impossible to cut the aluminum-foil substrate without bending it. For better reproducibility, the Vantablack absorbers should be grown directly on a substrate of the right size, such that unfolding of the vertically aligned CNT forest can be avoided. Similar sensitivity to substrate bending was not observed with other absorber materials.

### 3.4 The effect of acoustic carrier gas

The carrier gas comparisons for all investigated absorbers are presented in Fig. 10, as measured with the FTIR instrument. The wavenumber range of Fig. 10 corresponds to acoustic frequencies from 40.5–679 Hz (marked with an arrow in Fig. 4a). The shapes of the FTIR curves depend on the modulation-frequency dependencies, as discussed above. (Also, see Fig. 4b; the similarity with the curve of Fig. 10 is apparent). The smooth Nextel-painted surface benefits the most from the exchange of the carrier gas. The dips in the Nextel curve of Fig. 10 are caused by absorption peaks caused by water vapor. These peaks are not visible with other absorbers, because the higher SNR of measurement leads to better cancellation of absorption peaks when calculating the He/N_2_ signal ratio.

## 4 Conclusions

In conclusion, we have conducted an experimental comparison of photoacoustic responsivities of different highly absorptive materials. Most of the tested absorbers – two CNT absorbers and a Nextel-painted absorber – have nearly 100 % emissivities (Table 1) and look black when observed by naked eye. For example, the emissivity of Nextel-painted surface has been measured to be nearly constant 0.97 in the mid-infrared range, between 5 μm and 20 μm (500 cm^−1^ to 2000 cm^−1^) [26]. Despite the near-unity emissivities of the investigated absorber materials, the photoacoustic efficiencies vary significantly depending on the material, at least within the wavelength range (0.633 nm to 25 μm) and acoustic frequencies covered in our study. Self-made low-cost candle soot absorber was found to give the highest photoacoustic response within the entire spectral range, making it a good candidate for the future development of infrared power detectors. On the other hand, the PA responsivity of the candle soot absorber drops as a function of wavelength, closely following the spectral dependency of emissivity reported earlier [33]. Similar to their spectral emissivities, the CNT absorbers and Nextel have spectrally relatively flat PA responsivities [18,26,34]. The spray-coated CNT absorber (S-VIS) appears promising especially in the longer infrared; its potential for THz photoacoustic detectors should be investigated. In all cases, the PA signal can be maximized by a proper choice of the acoustic carrier gas. For example, with the candle-soot absorber, helium provides up to 80 % enhancement of the PA signal compared to nitrogen, as measured with typical modulation frequencies between 40 Hz and 120 Hz.

## Declaration of Competing Interest

The authors declare that they have no known competing financial interests or personal relationships that could have appeared to influence the work reported in this paper.

## Figures and Tables

**Table A1:** Laser wavelengths along with the corresponding wavenumbers and modulation frequencies.

λ [μm]	ν [cm^−1^]	f [Hz]
1.63	6135	621
3.39	2950	299
9.24	1087	110
14.85	673	68

## Appendix A

### The effect of modulation frequency

The FTIR ratios in Fig. 9 of the main article can be partly explained by the different modulation-frequency dependencies of different absorbers. These are exemplified below in Fig. A1, which shows the PA responses of the absorbers recorded at 14.85 μm wavelength and with modulation frequencies from 40 Hz to 650 Hz. The differences between different absorber types are clearly seen in Fig. A1b, where the modulation-frequency dependencies are compared to that of the candle soot absorber. In particular, the distinct differences in the modulation-frequency dependencies of Vantablack and candle soot are worth pointing out, as they explain the respective spectral shape in the FTIR measurement (Fig. 9 of the main article).

### FTIR measurements

As explained in the main article, the FTIR spectrometer converts each optical wavelength to a different modulation (acoustic) frequency. In order to validate the FTIR measurements with laser measurements (Fig. 9 of the main article), we adjusted the laser chopping frequencies at each wavelength such that they equal to the respective FTIR modulation frequencies. These modulation frequencies $f\;\left[\text{Hz}\right]$, were calculated with equation $f=2u\tilde{\nu }$, where u=0.05064 cm/s is the FTIR mirror scanner speed and $\tilde{\nu }\;\left[\text{c}{\text{m}}^{-1}\right]\;$is the wavenumber of light. (Note that this equation is equivalent to that shown in the main text for wavelength, since $\tilde{\nu }\;\left[\text{c}{\text{m}}^{-1}\right]=10000/\lambda \;\left[\text{μm}\right]$, where $\lambda \;\left[\text{μm}\right]$ is the wavelength). The modulation frequencies calculated for the lasers used in the validation are presented in Table A1.

The FTIR average spectra in Fig. 8 were calculated from 10 separate measurements of the same sample and each measurement was an average of 20 FTIR scans. All the measured spectra are presented below in Fig. A2. The spectral resolution in Fig. A2 of the Appendix and in Fig. 8, Fig. 9, Fig. 10 of the main article is 15 cm^−1^, which corresponds to a maximum mirror displacement of 0.667 mm and scan time of 1.32 s. The measurements were also used to calculate the standard deviations and the standard uncertainties plotted with shaded areas in Fig. 9.
